# e-Health Literacy Scale, Patient Attitudes, Medication Adherence, and Internal Locus of Control

**DOI:** 10.3928/24748307-20230417-01

**Published:** 2023-06

**Authors:** Donrie J. Purcell, Gesulla Cavanaugh, Kamilah B. Thomas-Purcell, Joshua Caballero, Drenna Waldrop, Victoria Ayala, Rosemary Davenport, Raymond L. Ownby

## Abstract

**Background:**

Health literacy is related to a variety of health outcomes, including disease control, health-related quality of life, and risk for death. Few studies have investigated the relation of electronic health literacy (e-health literacy) to outcomes or the mechanism by which they may be related.

**Methods:**

Secondary data were drawn from participants in a larger study on chronic disease self-management who were age 40 years and older, had at least one chronic health condition and a health literacy score of 8th grade or below on the validated short form of the Rapid Estimate of Adult Literacy in Medicine. Participants completed the e-Health Literacy Scale (eHEALS), the Multidimensional Health Locus of Control scale, a modified version of the Attitudes Toward Health Care Providers Scale (ATHCPS), the Wake Forest Physician Trust Scale (WFPTS), and the Gonzalez-Lu adherence questionnaire. Hypothesized relations were evaluated in a bootstrapped path analytic model using the Mplus statistical software.

**Key Results:**

Participants included 334 individuals (mean age: 57.5 years; 173 women and 161 men) with Black, Indigenous, and People of Color accounting for 83.3% of the participants and White individuals making up 16.7% of the participants. Model results showed that after controlling for age, education, gender, and race, the eHEALS score was significantly related to the ATHCPS and WFPTS but not to the Gonzalez-Lu adherence questionnaire (*p* < .05). The eHEALS score was significantly related to the Multidimensional Health Locus of Control scale. Analysis of indirect effects showed that a portion of the relation between e-health literacy and patient attitude and adherence was mediated by internal locus of control (all *p* < .05).

**Conclusions:**

In this study, e-health literacy was related to important patient attitude and behavior variables via locus of control. This finding has implications for the importance of improving patients' ability to use the internet to access and effectively use health information. [***HLRP: Health Literacy Research and Practice*. 2023;7(2):e80–e88.**]

## Plain Language Summary

Health literacy refers to how well people can understand and use health-related information to make informed decisions about their health. This study looked at how eHealth literacy (the ability to find and use health information online) is related to health outcomes and the ways in which they are connected. The study used data from people who were older than age 40 years, had at least one chronic health condition, and had a low level of health literacy. Participants completed surveys that measured eHealth literacy, attitudes toward health care providers, trust in physicians, medication adherence, and health locus of control (the belief that one's health is under their control or the control of external factors). The results of the study showed that eHealth literacy was related to attitudes toward health care providers and trust in physicians, but not to medication adherence. The study also found that eHealth literacy was related to internal health locus of control, meaning that people who had higher eHealth literacy were more likely to believe that they have control over their health outcomes. The study showed that the relationship between eHealth literacy and patient attitude and adherence was partially mediated by internal health locus of control. Overall, the study highlights the importance of improving patients' ability to use the internet to access and use health information. It suggests that eHealth literacy may be an important factor in improving patient attitudes toward health care providers and their trust in physicians, which may ultimately lead to better health outcomes.

Understanding why individuals make certain health decisions in the face of technology's growing influence on health literacy has been a perplexing problem and a goal of many researchers. Few studies have investigated the relationship between electronic health (eHealth) literacy and health outcomes, or the mechanisms by which they may be related. eHealth literacy is defined as the ability to seek, find, understand, and appraise health information from electronic sources (Norman & Skinner, 2006). Additionally, eHealth literacy uses emerging information and communication technology, especially the internet, to improve or enable health care (Eng, 2001). Fox and Fallows (2003) noted that among Americans with regular internet access (128 million people), 66% of them sought health and medical information. This number is greater than the number of physician office visits (2.27 million), and ambulatory care visits to hospital outpatient and emergency departments (2.75 million) combined (Fox & Rainie, 2000). Despite the positive attributes of health information retrieved from the internet, there may also be negative consequences for patients (Cline & Haynes, 2001), which include the effects on the patient-physician relation, participation in prevention and screening programs, and adherence to treatment (Tan & Goonawardene, 2017). Our goal is to understand if the relationship between eHealth literacy and the patient health decision-making process is mediated by the concept of internal locus of control as proposed by Rotter (1966). Allen (2017) argued that to better understand why or how a relationship between health outcome variables occur, mediation analysis is often used.

## Material and Methods

This study explored the existence of a relationship(s) between the electronic health literacy scale (eHEALS) and (a) trust in physicians as measured by the Wake Forest Physician Trust Scale (WFPTS); (b) attitudes toward providers as measured by the Attitudes Toward Health Care Providers Scale reworded to make it more relevant to persons with chronic health conditions; and (c) adherence to medication as measured by the Gonzalez-Lu adherence questionnaire among adults age 40 years and older suffering from one or more chronic diseases (e.g., high blood pressure, diabetes, cancer). Additionally, this study sought to investigate the role of the construct Internal Health Locus of Control (IHLOC) measured by the Multidimensional Health Locus of Control Scale as a mediating variable between eHEALS and (a) trust in physicians, (b) attitudes toward providers and, (c) adherence to medication.

### Measures of Interest

***Electronic health literacy.*** The eHEALS is currently the only instrument that aims to measure an individual's confidence in their ability to locate and evaluate online health information. It has been proven to be a valid and reliable measure of self-reported eHealth literacy among patients with chronic disease in the United States (Paige et al., 2017). The scale rates participants' skill level on 8 items using a 5-point Likert scale. The sum across the 8 equally weighted items is presented as a score out of 40 (Richtering et al., 2017). Initially, eHEALS focused principally on individuals age 25 years and younger (Norman & Skinner, 2006). It has been the conclusion of several studies that the instrument should be used among adult participants (i.e., age 40 years and older) because eHealth literacy is not well understood among this often overlooked and vulnerable group in online health communication and eHealth research (Paige et al., 2017; Tennant et al., 2015; Tse et al., 2008).

***Trust in physicians.*** Trust in one's physician is a central feature of the patient-physician relationship (Pearson & Raeke, 2000). The WFPTS was developed by Hall et al. (2002) to measure levels of patient trust in primary care providers. The WFPTS asks participants to indicate their trust in their physician on 10 items reversed scored on a 5-point-Likert scale from *totally agree* = 1, to *totally disagree* = 5. The advantage of using the WFPTS is that it has good internal consistency (alpha = .93), a good test-retest reliability (*r* = 0.75) and the distribution of the questionnaires are less skewed than other questionnaires (Hall et al., 2002).

***Attitudes toward providers.*** The Attitudes Toward Healthcare Provider Scale (ATHCPS) was used to measure the participants' attitude toward their providers. This is a 19-item scale that measures patients' attitudes toward providers. Individual items from the ATHCPS were scored using a 6-point Likert-style rating system ranging from *strongly agree* to *strongly disagree* (Bodenlos et al., 2004). Individual items from the ATHCPS were scored using a 6-point Likert scale ranging from *strongly agree* to *strongly disagree* (Bodenlos et al., 2004). The advantage of using the WFPTS is that it has good internal consistency (alpha = .93), a good test-retest reliability (*r* = 0.75), and the questionnaire distributions are less skewed than other questionnaires (Hall et al., 2002). Several studies have suggested that patients' attitudes toward their health care providers affect certain health behaviors, including adherence to medication.

***Adherence to medication.*** Medication adherence is the extent to which a patient's behavior (e.g., taking medications with respect to timing, dosage, and frequency) corresponds with recommendations from a health care provider (Vrijens et al., 2012).

The Gonzalez-Lu are self-reported medication adherences questions developed by Lu et al. (2008), which uses a six-step scale ranging from *very poor* to *excellent*, has been validated in patients who are HIV positive, and it measures patients' average ability to take their medication as prescribed. These questions were further validated by Gonzalez et al. (2013) study with small variations.

Two questions addressing medication adherence were selected from each study. Questions 1 and 3 examined medication adherence in terms of the percentage of time patients took their medication as prescribed by their doctor, ranging from 0% to 100% on a weekly and monthly basis, respectively. Questions 2 and 4 used a 5-point Likert scale ranging from 0 = *very poor* to 5 = *excellent* to address medication adherence in the last week or month. Lu et al. (2008) and Gonzalez et al. (2013) noted that qualitative self-ratings were more accurate self-reports in predicting Medication Event Monitoring System adherence. Gonzalez et al. (2013) supported the validity of this easily administered self-report measure in assessing medication adherence among adults with type 2 diabetes. According to Sidorkiewicz et al. (2016), the scale showed good temporal stability (test-retest); there was good convergent validity with the Morisky Medication Adherence Scale of 4 items (Morisky et al., 1986).

***Internal health locus of control.*** Locus of control, an aspect of social learning theory, describes how individuals view their relationship to the environment (Rotter, 1966). Locus of control indicates the extent to which individuals believe that they can control certain outcomes (i.e., level of trust in physicians, attitudes toward providers, and adherence to medication) that affect them (Rotter, 1966). This has been one of the most effective measures of health-related beliefs for more than a quarter of a century (Moshki et al., 2007). The Multidimensional Health Locus of Control scale includes 18 items and consists of 3 sub-scales, namely IHLOC, Powerful Others Health Locus of Control, and Chance Health Locus of Control (Moshki et al., 2007). Each of these subscales contains 6 items with a 6-point Likert response scale ranging from *strongly agree* to *strongly disagree*. Scales are scored by summing respective items for a total scale score (Wallston et al., 1978). Alpha reliabilities for the Multidimensional Health Locus of Control (6-item scales forms) ranged from .673 to .767 and, when forms A and B combined to make 12-item scales, the alpha reliabilities increased (.830 to .859). An individual with an internal locus of control believes that outcomes (i.e., health outcomes) are a direct result of their own behavior (Wallston & Wallston, 1982). According to MacKinnon (2011), the use of a mediating variable (for example, IHLOC) to help understand relations between patient factors and health outcomes may produce practical and theoretical information that leads to useful interventions. This conceptual approach has received considerable support in the literature (Chen, 1990; Lipsey, 1993; Sidani & Sechrest, 1999).

### Study Design

This was a cross-sectional study design that used data drawn from a larger study entitled *Fostering Literacy for Good Health Today* (FLIGHT) (Ownby et al., 2017) within Broward and the surrounding counties of Florida and Atlanta, GA. FLIGHT investigated whether a computer-delivered tailored intervention targeting health literacy could be deployed either as an information kiosk in a clinical office or on the internet and be cost-effective in improving patients' health literacy and adherence. The inclusion criteria for this study were individuals age 40 years and older taking medication for one or more chronic disease with a low health literacy score (19 or less) as verified by the Rapid Estimate of Adult Literacy in Medicine 20 (REALM 20). REALM 20 is a 20-word recognition and pronunciation test (based on the REALM with 66 words) to provide clinicians with a quick and valid assessment of patient health literacy. It uses a validated score from previous cut-off settings of the REALM to maximize the sensitivity of the screening procedure and demonstrate the presence of low health literacy defined as a reading level less than 8th grade (Han et al., 2017). Participants age 40 years and older who graduated from college were excluded from the original study as this population was unlikely to have low health literacy (Ownby et al., 2017). Also, Seo et al. (2017) showed that they tend to have a low prevalence of chronic diseases.

This study was approved by the Institutional Review Boards of Nova Southeastern University and Emory University. Variables of interest such as age, gender, race and ethnicity, education, and income were investigated as potential confounders in the relationships of interest. The independent variable was the eHEALS. The dependent variables were (a) trust in physician as collected by the WFPTS, (b) attitude toward providers as collected by using a modified version of ATHCPS, (c) and adherence to medication as collected from the Gonzalez-Lu adherence questionnaire. The mediating variable of interest, IHLOC, was collected using the Multidimensional Health Locus of Control Scales. These self-report measures were administered via automated computer administered self-interview software.

**Figure 1.**
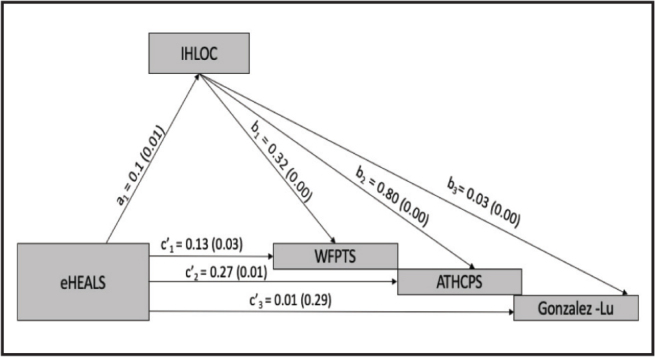
Regression coefficients and p values for the relationships between eHEALS, IHLOC, WFPTS, ATHCPS, and Gonzalez-Lu adherence questionnaire. Note. Analysis conducted in Mplus with full-information maximum likelihood. ATHCPS = Attitudes Toward Health Care Providers Scale; eHEALS = e-Health Literacy Scale; IHLOC = Internal Health Locus of Control; WFPTS = Wake Forest Physician Trust Scale.

Sample size was determined for key hypotheses in the larger study *a priori* using the random effects procedure in PASS 16 (NCSS Statistical Package for Social Sciences, Kaysville UT). SPSS Version 27 were used to calculate the internal reliability of the scales used in the study. MPlus version 8.4 with using full-information maximum likelihood (FIML) estimation was used for tests to study the hypotheses that relationships between eHEALS and WFPTS, ATHCPS, and the Gonzalez-Lu adherence questionnaire are mediated by IHLOC. Bias-corrected bootstrapping was used for tests of direct and indirect effects using 5,000 iterations. Statistical significance was determined at the 95% confidence level for the statistical tests used to generate the study outcome. Frequency and descriptive statistics were calculated for the variables collected. As the Gonzalez-Lu adherence questionnaire comprised four questions, we calculated scores from a principal factors analysis composite providing an item-weighted measure of medication adherence in *Z* score form (**Table 1**). Multivariate regression coefficients were used to identify and calculate the direct and indirect paths of the model (**Figure 1**).

**Table 1:**
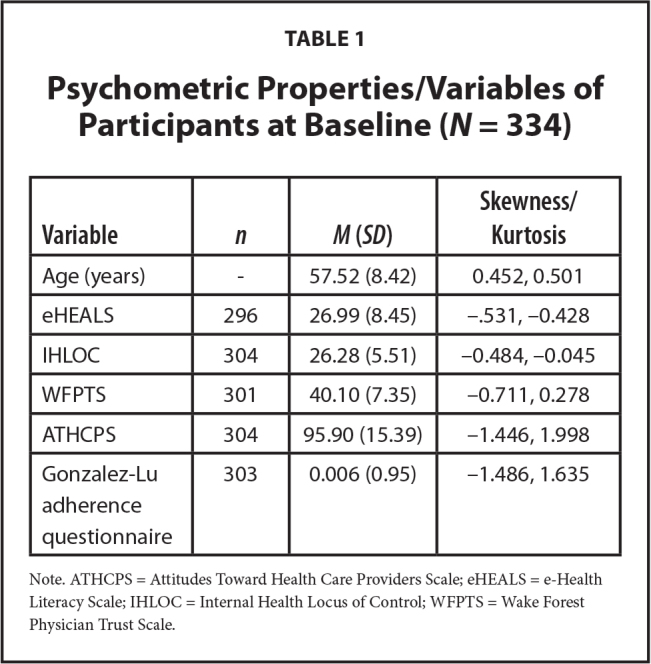
Psychometric Properties/Variables of Participants at Baseline (*N* = 334)

**Variable**	***n***	***M* (*SD*)**	**Skewness/Kurtosis**
Age (years)	-	57.52 (8.42)	0.452, 0.501
eHEALS	296	26.99 (8.45)	−.531, −0.428
IHLOC	304	26.28 (5.51)	−0.484, ‒0.045
WFPTS	301	40.10 (7.35)	−0.711, 0.278
ATHCPS	304	95.90 (15.39)	−1.446, 1.998
Gonzalez-Lu adherence questionnaire	303	0.006 (0.95)	−1.486, 1.635

Note. ATHCPS = Attitudes Toward Health Care Providers Scale; eHEALS = e-Health Literacy Scale; IHLOC = Internal Health Locus of Control; WFPTS = Wake Forest Physician Trust Scale.

## Results

The sample consisted of 334 participants of whom 48% (*n* = 161) were men and 49% (*n* = 173) were women. The mean age of the participants was 57.4 years (standard deviation [*SD*] = 8.42) years. Most of the participants (65.4%, *n* = 219) had attained 12 years of education or less, and 54% (*n* = 180) earned less than $10,000 annually. Black, Indigenous, and People of Color accounted for 83.3% of participants and White people represented 16.7% of the participants. Descriptive statistics for study measures are detailed in **Table 1**.

**Table 2:**
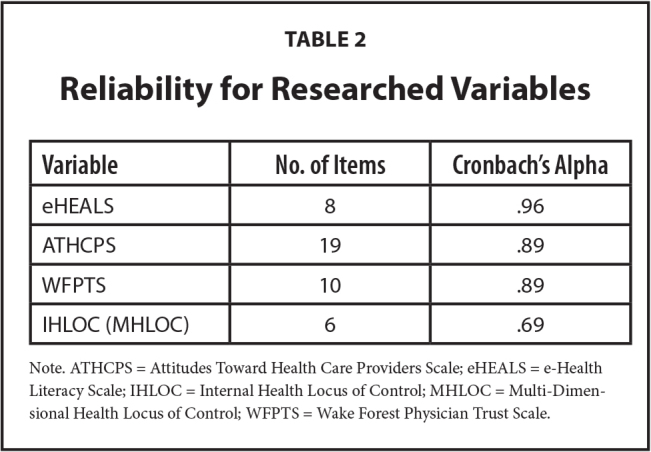
Reliability for Researched Variables

**Variable**	**No. of Items**	**Cronbach's Alpha**
eHEALS	8	.96
ATHCPS	19	.89
WFPTS	10	.89
IHLOC (MHLOC)	6	.69

Note. ATHCPS = Attitudes Toward Health Care Providers Scale; eHEALS = e-Health Literacy Scale; IHLOC = Internal Health Locus of Control; MHLOC = Multi-Dimensional Health Locus of Control; WFPTS = Wake Forest Physician Trust Scale.

Additionally, the internal reliability of the scales used in this study was calculated, and the results are presented in **Table 2**.

Considering missing data for some variables due to computer failure, we used FIML estimation in model estimation as this strategy is effective in creating unbiased estimates in the presence of missing data (Enders & Bandalos, 2001).

### Mediation Effects

The results of the regression coefficients a1, b1, b2, and b3 of the path model represented the indirect effect of the eHEALS via IHLOC on the outcome variables: (a) trust in physicians, (b) attitudes toward providers, and (c) adherence to medication (**Figure 1**). Similarly, the results of regression coefficients c1, c2, and c3 represented the direct effect of eHEALS on (a) trust in physicians, (b) attitudes toward providers, and (c) adherence to medication, respectively (**Figure 1**).

The results of the path model showed that the eHEALS were also positively related to IHLOC. Similarly, the eHEALS was positively related to WFPTS and ATHCPS. Further, IHLOC was positively related to WFPTS, ATHCPS and the Gonzalez-Lu adherence questionnaire (**Table 3**).

**Table 3:**
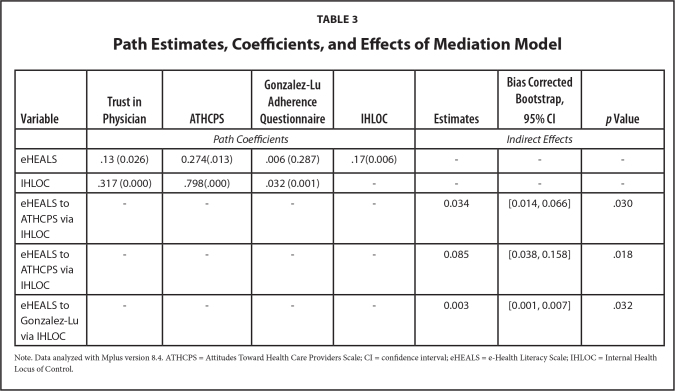
Path Estimates, Coefficients, and Effects of Mediation Model

**Variable**	**Trust in Physician**	**ATHCPS**	**Gonzalez-Lu Adherence Questionnaire**	**IHLOC**	**Estimates**	**Bias Corrected Bootstrap, 95% CI**	***p* Value**
	*Path Coefficients*				*Indirect Effects*		
eHEALS	.13 (0.026)	0.274(.013)	.006 (0.287)	.17(0.006)	-	-	-
IHLOC	.317 (0.000)	.798(.000)	.032 (0.001)	-	-	-	-
eHEALS to ATHCPS via IHLOC	-	-	-	-	0.034	[0.014, 0.066]	.030
eHEALS to ATHCPS via IHLOC	-	-	-	-	0.085	[0.038, 0.158]	.018
eHEALS to Gonzalez-Lu via IHLOC	-	-	-	-	0.003	[0.001, 0.007]	.032

Note. Data analyzed with Mplus version 8.4. ATHCPS = Attitudes Toward Health Care Providers Scale; CI = confidence interval; eHEALS = e-Health Literacy Scale; IHLOC = Internal Health Locus of Control.

The results of the model revealed that the indirect effects of IHLOC between several model elements were statistically significant: (a) eHEALS and WFPTS (b) eHEALS and ATHCPS and (c) eHEALS and Gonzalez-Lu adherence questionnaire (**Table 2**). These results established that internal health locus of control mediated the relationship between trust in physicians, attitudes toward providers, and adherence to medication (**Figure 1**). The regression of the eHEALS on the covariates of interest (i.e., race and ethnicity, age, gender, education, and income) showed that the variables age and education were positively and significantly related to the eHEALS (**Table 4**).

**Table 4:**
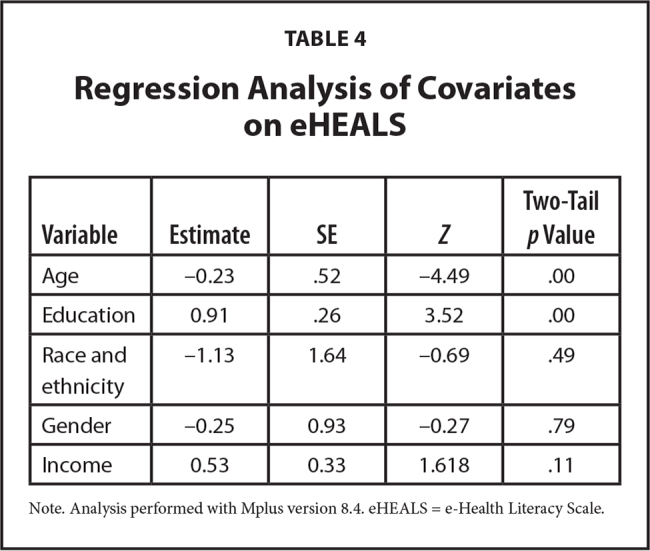
Regression Analysis of Covariates on eHEALS

**Variable**	**Estimate**	**SE**	***Z***	**Two-Tail *p* Value**
Age	−0.23	.52	−4.49	.00
Education	0.91	.26	3.52	.00
Race and ethnicity	−1.13	1.64	−0.69	.49
Gender	−0.25	0.93	−0.27	.79
Income	0.53	0.33	1.618	.11

Note. Analysis performed with Mplus version 8.4. eHEALS = e-Health Literacy Scale.

### Model Fit

Path analysis via structural equation models (SEM) allows for testing of direct and indirect relations between variables based on a hypothesized model. Goodness-of-fit indexes provide an assessment of how well the hypothesized model fits empirical data (Phan, 2009). Initial results of the SEM path model suggested a significant lack of fit, with a significant chi-square value, root mean squared error of approximation greater than a desirable value, and fit indexes less than 0.90. Explorations of reasons for lack of model fit suggested that race was significantly related to IHLOC, and that income, education, and race were significantly related to each other. We modified the SEM model to include these relations, although they were not initially hypothesized. After including these relations, adequate model fit was achieved (**Table 5**).

**Table 5:**
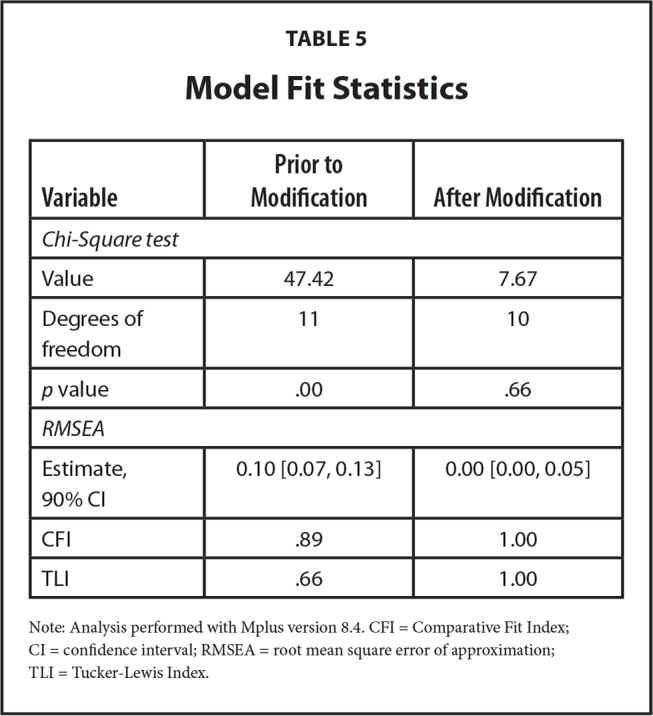
Model Fit Statistics

**Variable**	**Prior to Modification**	**After Modification**
*Chi-Square test*		
Value	47.42	7.67
Degrees of freedom	11	10
*p* value	.00	.66
*RMSEA*		
Estimate, 90% CI	0.10 [0.07, 0.13]	0.00 [0.00, 0.05]
CFI	.89	1.00
TLI	.66	1.00

Note: Analysis performed with Mplus version 8.4. CFI = Comparative Fit Index; CI = confidence interval; RMSEA = root mean square error of approximation; TLI = Tucker-Lewis Index.

## Discussion

This study is novel in its review of the IHLOC as a mediating variable in the relationship between the eHEALS and WFPTS, and ATHCPS and the Gonzalez-Lu adherence questionnaire among individuals age 40 years and older. The mean and *SD* of the eHEALS in this study were similar to the results of Paige et al. (2017) whose study population similarly suffered from at least one chronic disease, and that of Britt and Hatten (2013) that included primarily university undergraduate students. This illustrated that the eHEALS functions well as an instrument for measuring eHealth literacy among different age groups regardless of a person's disease attributes. The investigators of this study agree with the Nunnally & Bernstein (1994) assessment that an Cronbach's alpha of .7 should be considered as evidence of good reliability. As an exploratory study, the investigators of this study concur with Hair et al. (2010) that values as low as 0.60 are acceptable for exploratory research. Trust between a physician and a patient is important (Li et al., 2016), and accessing online health information helps patients to effectively communicate with their doctors (Peng et al., 2019). Chronic disease patients use the internet to find information on medications, nutrition, disease management, and disease prevention (Shiferaw et al., 2020). Our findings showed that IHLOC mediated the relationships between eHEALS and a participant's trust in physicians, their attitudes toward providers, and adherence to medication. Consequently, we proposed an intervention such as personal computers, tablets with videos, or interactive self-help to tools, which serve to improve underlying health literacy skills, such as the ability to read. These approaches can be delivered by various health care providers, clinic staff, and public health professionals (Jacobs et al., 2014). Notably, many of the current Healthy People 2030 objectives place a specific focus on health communication and eHealth information, which supports the importance of public health policies focused on improving eHealth literacy across the United States (National Institutes of Health, 2021).

Our findings of a positive relationship between the eHEALS and WFPTS contribute to the current body of empirical evidence on this issue. We argue that patients should be cautious when using online information for health purposes as the practice can result in inappropriate requests for clinical interventions and compromise one's health (Eysenbach & Köhler, 2002). A study by Hu et al. (2012), showed that an increased number of adult patients are seeking health-related information via the internet prior to their doctor visits, and previous research by Sillence et al. (2007) also showed patients with high electronic literacy rates tend to visit the web before consulting with their physicians.

Our findings of a positive relation between patient eHEALS and ATHCPS suggest that health information found on the web offers an opportunity to improve the patient-physician relationship. This may be accomplished by a shared burden of responsibility for health knowledge (Gerber & Eiser, 2001), and the general enhancement of communication between the patient and provider (Robinson et al., 1999). Consistent with previous research by Kim et al. (2018), we acknowledge that several variables such as education and age may have concomitantly contributed to patients' attitudes toward their providers along with their eHEALS score.

Research on the effect of the internet on medication adherence is rare (Im & Huh, 2017), and even more so research focused on understanding the role of health LOC in the relationship. Im and Huh (2017) noted that the interplay between patients' online information seeking behavior, beliefs about medication, and medication adherence warrants further research. The findings of this study suggest that a person's IHLOC successfully mediated an indirect relationship between eHealth literacy and adherence to medication. In other words, having a high eHealth literacy score meant that a participant likely knew that they should take their medication. Without a sufficient IHLOC, it can be speculated that the participants would be less likely to adhere to medication recommendations. This is not unlike the study by Náfrádi et al. (2017), which noted that patient self-empowerment or health locus of control can promote medication adherence. Conversely, another study by Im and Huh (2017) found an increased frequency of patient online-seeking behavior was positively related to nonadherence.

## Study Limitations

Data were cross-sectional, only reflecting a snapshot in time or the period from 2016 to 2019 and generalizability of the findings cannot be achieved. Also, because this was a cross-sectional study design, causal inferences cannot be drawn from the results. It is entirely plausible there may have been a high risk of recall bias in this study as participants gave self-reports of memories, which may have been influenced by subsequent events and experiences. Missing data was another limitation. However, we were able to address this issue using Mplus version 8.4 with FIML estimation, which allowed the authors to use all the available data, even when bootstrapping, without having to lose vital data points in the process of analysis. This ultimately allowed for the validity of the analysis to be sounder and more rigorous.

## Study Strengths

Despite the limitations, there were many strengths to this study. One of the key strengths was the ability to show which of the operationalized variables were associated with eHEALS in the sample population. Conversely, other studies on mediation analysis have relied on other statistical packages to conduct the analysis that may not have dealt effectively with missing data. This study provides an evidence-based foundation to answering questions about how electronic health literacy, when mediated by IHLOC, can affect medication adherence, trust in one's provider, and consequent attitude toward the provider, which has implications for patient-provider interactions in the clinical setting, engaging patients in their own care and improving health outcomes.

## Conclusion

This study was conducted to contribute to current research on how eHealth literacy influences health attitudes and behaviors such as trust in physicians, attitudes toward health care providers, and medication adherence via a mediating variable—IHLOC. These findings have implications for research aimed at improving patient-provider communications through programs and policies that can increase patients' efficacy in using the internet to access health information. Future research should include qualitative studies that allow for a more in-depth understanding of the personal choices of adults (age 40 years and older) who make use of internet technology for health purposes. Additionally, longitudinal studies are needed to produce more reliable inferences about directionality and associations between the factors that may influence the study outcomes over time.
